# Chromosome-scale haplotype-phased genome assemblies of the male and female lines of wild asparagus (*Asparagus kiusianus*), a dioecious plant species

**DOI:** 10.1093/dnares/dsac002

**Published:** 2022-01-18

**Authors:** Kenta Shirasawa, Saki Ueta, Kyoko Murakami, Mostafa Abdelrahman, Akira Kanno, Sachiko Isobe

**Affiliations:** 1 Kazusa DNA Research Institute, Kisarazu, Chiba 292-0818, Japan; 2 Kagawa Prefectural Agricultural Experiment Station, Ayagawa, Kagawa 761-2306, Japan; 3 Graduate School of Life Sciences, Tohoku University, Sendai, Miyagi 980-8577, Japan; 4 Biotechnology Program, Faculty of Science, Galala University, Suze, Galala 43511, Egypt; 5 Botany Department, Faculty of Science, Aswan University, Aswan 81528, Egypt

**Keywords:** dioecy, genome assembly, haplotype phasing sequence, linked reads, structural variation

## Abstract

*Asparagus kiusianus* is a disease-resistant dioecious plant species and a wild relative of garden asparagus (*Asparagus officinalis*). To enhance *A. kiusianus* genomic resources, advance plant science, and facilitate asparagus breeding, we determined the genome sequences of the male and female lines of *A. kiusianus*. Genome sequence reads obtained with a linked-read technology were assembled into four haplotype-phased contig sequences (∼1.6 Gb each) for the male and female lines. The contig sequences were aligned onto the chromosome sequences of garden asparagus to construct pseudomolecule sequences. Approximately 55,000 potential protein-encoding genes were predicted in each genome assembly, and ∼70% of the genome sequence was annotated as repetitive. Comparative analysis of the genomes of the two species revealed structural and sequence variants between the two species as well as between the male and female lines of each species. Genes with high sequence similarity with the male-specific sex determinant gene in *A. officinalis*, *MSE1*/*AoMYB35*/*AspTDF1*, were presented in the genomes of the male line but absent from the female genome assemblies. Overall, the genome sequence assemblies, gene sequences, and structural and sequence variants determined in this study will reveal the genetic mechanisms underlying sexual differentiation in plants, and will accelerate disease-resistance breeding in garden asparagus.

## 1. Introduction

*Asparagus kiusianus* is a wild relative of garden asparagus (*Asparagus officinalis*). While garden asparagus is a cultivated species belonging to the Asparagaceae family and is consumed as a vegetable crop around the world, *A. kiusianus* is native to the coastal regions of Japan.^1^ Therefore, *A. kiusianus* might exhibit tolerances and/or resilience to abiotic and biotic stresses. Although *A. kiusianus* has been identified as a potential donor of stem-blight disease resistance in asparagus breeding programs,^2^ neither the genetic mode nor the genetic loci of resistance have been elucidated to date.

*Asparagus* *officinalis* is a dioecious species and is widely recognized as a model for sex determination in plants. Recent studies indicate that the male-specific *MYB*-like gene, *MSE1*/*AoMYB35*/*AspTDF1*, located at the masculinization-promoting *M* locus of the Y-specific region in asparagus, functions in sex determination in asparagus.^3–5^ Since *A. kiusianus* is also a dioecious plant species, like garden asparagus, it is possible that both species share the same system of sex determination. Therefore, comparative genome sequence and structure analyses between the two species could provide insights into the molecular mechanisms underlying sex determination in Asparagaceae and the evolutionary processes involved therein.

Advances in sequencing technologies have enabled the whole-genome sequencing of various plant species, thus providing fundamental information required for understanding the plant biology and accelerating breeding programs. Nevertheless, while the genome sequence data of garden asparagus^3^ and transcriptome data of *A. kiusianus*^6^^,^^7^ have been made publicly available, no whole-genome sequence data have been released for *A. kiusianus* to date. Owing to the dioecious nature of *A. kiusianus*, which leads to allogamy, its genome is predicted to be highly heterozygous. Therefore, haplotype-phased genome sequence data would be useful for dissecting the allelic sequence and structural variations in *A. kiusianus*. In this study, we employed a linked-read technology (10X Genomics, Pleasanton, CA, USA) to construct haplotype-based genome sequence assemblies of the male and female lines of *A. kiusianus*. The genome sequence assemblies were then used for gene prediction and sequence and structural variant discovery. Overall, the genome sequence information of *A. kiusianus* obtained in this study could accelerate studies on plant sex determination and facilitate asparagus breeding programs.

## 2. Materials and methods

### 2.1. Plant materials

Male (K1) and female (K2) lines of *A. kiusianus* cultivated at Kagawa Prefectural Agricultural Experiment Station (Kagawa, Japan) were used in this study. Genomic DNA was extracted from the stems of young seedlings using the modified cetyltrimethylammonium bromide method.^8^

### 2.2. Genome sequencing and assembly

Genomic DNA libraries of male and female lines were prepared using the Chromium Genome Library Kit v2 (10× Genomics), and sequenced on NovaSeq 6000 (Illumina, San Diego, CA, USA) in paired-end, 150 bp mode. The sequence reads were assembled with Supernova (10× Genomics) to construct the contig sequences, scaffold the contigs, and resolve haplotype phases. DNA library preparation, sequencing, and assembly were conducted by Takara Bio (Shiga, Japan) as an outsourcing service.

The genome sizes of male and female lines were estimated based on short reads using Jellyfish. To construct pseudomolecule sequences at the chromosome level, the assembled contigs were aligned against the sequence of 10 *A. officinalis* chromosomes (reference) using RaGoo.

The software tools used for data analyses are listed in Supplementary Table S1.

### 2.3. Repetitive sequence analysis and gene prediction

Repetitive sequences in the assemblies were identified with RepeatMasker, using repeat sequences registered in Repbase and a *de novo* repeat library built with RepeatModeler.

RNA-Seq reads of *A. kiusianus* and *A. officinalis* were obtained from a public DNA database (GenBank Sequence Read Archive accession number: SRA1003110).^6^^,^^7^ The RNA-Seq reads, from which adapter sequences were trimmed with fastx_clipper in the FASTX-Toolkit, were aligned against the assembled sequences with HISAT2. Gene prediction was performed with BREAKER2 using the positional information of the repeats, RNAs, and peptide sequences of the predicted genes of *A. officinalis* (V1.1)^3^ released in Phytozome.

### 2.4. Comparative genome structure analysis of *Asparagus kiusianus* and *Asparagus officinalis*

Chromosome-level genome sequence assemblies of *A. kiusianus* (this study) and *A. officinalis* (V1.1)^3^ were compared with Minimap2, and the resultant Pairwise mApping Format files were visualized with pafr.

## 3. Results and data description

### 3.1. Haplotype-phased genome assembly

**Figure 1:**
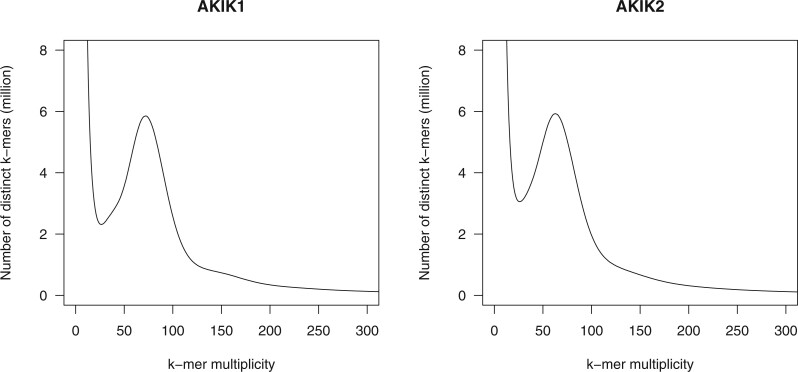
Estimation of the genome sizes of the male and female lines of *A. kiusianus*, based on *k*-mer analysis (*k *=* *17) with the given multiplicity values.

Short-read sequences of the male (143.7 Gb) and female (140.0 Gb) lines of *A. kiusianus* were obtained in this study, and their genome sizes were estimated at 1,563.8 Mb and 1,729.4 Mb, respectively (Fig. 1).

The short-read sequences of the male line were assembled into raw contigs (total length =3,724.6 Mb, N50 = 7.5 kb), which included gaps and all homologous sequences of the diploid genome (Supplementary Table S2). Then, the homologous sequences were flattened, and the gaps were filled by joining the sequence to its flanking sequence, thus producing megabubble sequences (total length =1,811.6 Mb, N50 = 170.6 kb) (Supplementary Table S2). Finally, two haplotype-phased genome assemblies (each containing 111,443 sequences) were generated from the megabubble sequences (Table 1). Haplotype 1 spanned 1,618.9 Mb in total with an N50 value of 155.5 kb, while haplotype 2 spanned 1,618.5 Mb in total with an N50 length of 155.3 kb. Complete Benchmarking Single-Copy Orthologs (BUSCO) scores were 88.4% and 88.6% for haplotypes 1 and 2, respectively (Table 1). The male genome assemblies for haplotype 1 and 2 were designated as AKIK1p1 and AKIK1p2, respectively.

**Table 1:** Statistics of the genome assemblies of the male and female lines of *A. kiusianus*

	AKIK1p1	AKIK1p2	AKIK2p1	AKIK2p2
Total contig size (bp)	1,618,866,837	1,618,519,636	1,567,596,515	1,567,276,205
Number of sequences	111,443	111,443	125,241	125,241
Sequence N50 length (bp)	155,490	155,269	59,656	59,610
Longest sequence size (bp)	2,375,321	2,375,321	1,231,068	1,222,162
Gap size (bp)	168,294,890	168,291,350	132,162,460	132,161,290
Single-copy complete BUSCOs	82.4%	82.6%	83.5%	83.4%
Duplicated complete BUSCOs	6.0%	6.0%	5.2%	5.2%
Fragmented BUSCOs	5.8%	5.5%	5.6%	5.6%
Missing BUSCOs	5.8%	5.9%	5.7%	5.8%

Similarly, short-read sequences of the female line were assembled into raw contigs (total length =3,780.7 Mb, N50 = 7.0 kb) and megabubble sequences (total length =1,703.6 Mb, N50 = 76.2 kb) (Supplementary Table S2). The resultant haplotype-phased assemblies (each containing 125,241 sequences) spanned 1,567.6 Mb in total with an N50 value of 59.7 kb for ‘haplotype 1’, and 125,241 sequences of 1,567.3 Mb in total with an N50 value of 59.6 kb for ‘haplotype 2’ (Table 1). Complete BUSCO scores were 88.7% and 88.6% for haplotypes 1 and 2, respectively (Table 1). The female genome assemblies for haplotype 1 and 2 were designated as AKIK2p1 and AKIK2p2, respectively.

The four sets of genome sequence assemblies of *A. kiusianus* (haplotypes 1 and 2 of male and female lines) were aligned against the chromosome-scale genome assembly of *A. officinalis*. A total of 96,224 sequences (1,535.9 Mb) for haplotype 1 and 96,224 sequences for haplotype 2 (1,535.3 Mb) in the male line, and 107,875 sequences (1,491.7 Mb) for haplotype 1 and 107,864 sequences (1,489.7 Mb) for haplotype 2 in the female line, could be aligned to the 10 chromosome sequences of *A. officinalis* (Table 2). Complete BUSCO scores ranged from 91.2% (AKIK1p1) to 91.8% (AKIK2p2). The nomenclature of the pseudomolecule sequences was based on the chromosome names of *A. officinalis* (ch01–ch10), where the chromosome 1 is the sex chromosome.^3^ Sequences that were unassigned to the *A. officinalis* genome were connected and termed chromosome 0 (ch00).

**Table 2:** Statistics of the pseudomolecule sequences of *A. kiusianus*

	AKIK1p1				AKIK1p2			
	Total length (bp)	Gap (%)	Number of contigs	Number of genes	Total length (bp)	Gap (%)	Number of contigs	Number of genes
ch01 (Y)	179,686,458	11.8	10,537	6,938	179,663,709	11.8	10,543	6,706
ch02	94,379,980	12.5	6,056	3,631	94,286,185	12.5	6,066	3,427
ch03	160,690,220	10.4	10,792	6,135	161,086,385	10.5	10,791	5,855
ch04	222,888,549	11.1	13,146	7,547	222,850,935	11.1	13,139	7,251
ch05	175,875,079	12.0	10,573	6,878	176,035,479	12.0	10,582	6,668
ch06	112,254,750	9.4	7,553	3,696	112,182,592	9.4	7,553	3,504
ch07	231,128,771	11.0	13,973	8,285	230,178,869	11.0	13,928	7,953
ch08	209,954,455	10.5	13,763	7,167	209,720,314	10.5	13,796	6,882
ch09	73,375,337	10.6	4,435	2,759	73,241,043	10.7	4,437	2,638
ch10	85,316,880	10.8	5,396	3,353	85,678,944	10.9	5,389	3,229
**Subtotal**	**1,545,550,479**	**11.0**	**96,224**	**56,389**	**1,544,924,455**	**11.0**	**96,224**	**54,113**
ch00	84,496,458	10.4	15,223	2,819	84,775,281	10.6	15,223	2,593
**Total**	**1,630,046,937**	**11.0**	**111,447**	**59,208**	**1,629,699,736**	**11.0**	**111,447**	**56,706**

	AKIK2p1				AKIK2p2			
		
	Total length (bp)	Gap (%)	Number of contigs	Number of genes	Total length (bp)	Gap (%)	Number of contigs	Number of genes

ch01 (X)	167,748,676	9.5	11,500	6,772	167,821,022	9.5	11,527	6,835
ch02	88,515,862	10.3	6,726	3,557	87,905,216	10.2	6,698	3,651
ch03	159,691,030	9.0	12,193	5,956	159,408,608	9.0	12,181	6,030
ch04	217,008,733	9.0	15,036	7,377	217,068,856	9.0	15,080	7,655
ch05	172,460,497	9.9	11,974	6,762	171,835,019	9.9	11,943	6,970
ch06	107,102,574	8.1	8,296	3,565	107,106,266	8.1	8,278	3,621
ch07	222,804,915	9.2	15,815	7,998	221,855,507	9.2	15,811	8,158
ch08	213,448,917	8.6	15,408	7,141	213,032,411	8.6	15,401	7,250
ch09	71,157,753	9.4	5,055	2,517	71,672,067	9.5	5,054	2,573
ch10	82,583,459	9.3	5,872	3,262	82,780,279	9.2	5,891	3,270
**Subtotal**	**1,502,522,416**	**9.2**	**107,875**	**54,907**	**1,500,485,251**	**9.2**	**107,864**	**56,013**
ch00	77,633,199	8.8	17,366	2,616	79,350,054	8.9	17,377	2,681
**Total**	**1,580,155,615**	**9.2**	**125,241**	**57,523**	**1,579,835,305**	**9.2**	**125,241**	**58,694**

Bold indicates subtotal (ch01 to ch10) and total values (ch01 to ch10 and ch00).

### 3.2. Repetitive sequence analysis and gene prediction

Repeat sequences occupied 66.8% (AKIK1p1), 66.8% (AKIK1p2), 68.1% (AKIK2p1), and 68.1% (AKIK2p2). The most abundant repetitive sequences were long terminal repeats (LTRs) (45.4–46.3%), followed by unclassified repeats (14.9–13.7%) and DNA transposons (4.7–4.8%) (Table 3).

**Table 3:** Repetitive sequences in the *A. kiusianus* genomes

Repeat type	AKIK1p1			AKIK1p2			AKIK2p1			AKIK2p2		
	Number of elements	Length occupied (bp)	%	Number of elements	Length occupied (bp)	%	Number of elements	Length occupied (bp)	%	Number of elements	Length occupied (bp)	%
SINEs	296	100,129	0.0	295	100,876	0.0	260	88,942	0.0	269	88,784	0.0
LINEs	36,139	22,836,398	1.4	36,171	22,868,973	1.4	36,022	22,848,619	1.5	36,257	22,865,141	1.5
LTR elements	534,586	740,043,346	45.4	534,419	739,629,919	45.4	538,917	731,783,197	46.3	538,772	731,702,512	46.3
DNA transposons	119,624	77,129,754	4.7	119,369	77,003,389	4.7	117,911	76,193,533	4.8	118,264	76,216,494	4.8
Unclassified	426,795	242,401,476	14.9	426,435	242,359,956	14.9	422,640	239,811,052	15.2	422,494	239,544,071	15.2
Small RNA	447	231,343	0.0	451	233,377	0.0	435	232,256	0.0	442	228,647	0.0
Satellites	1,945	843,970	0.1	1,894	847,909	0.1	2,155	901,555	0.1	2,171	901,844	0.1
Simple repeats	197,902	17,199,114	1.1	198,009	17,248,715	1.1	195,639	16,992,727	1.1	195,634	17,024,257	1.1
Low complexity	32,476	1,822,571	0.1	32,548	1,822,378	0.1	32,388	1,816,175	0.1	32,482	1,817,483	0.1

A total of 404.2 million RNA reads for 18 samples were mapped to the genome sequences. The mapping rates of *A. kiusianus* RNA-Seq reads were 93.7–94.1%, while those of *A. officinalis* reads were 82.4–82.6%. Based on the positions of RNA-Seq reads on the genome sequences, a total of 59,208, 56,706, 57,523, and 58,694 potential protein-coding genes were predicted in AKIK1p1, AKIK1p2, AKIK2p1, and AKIK2p2, respectively (Table 2), of which 365, 380, 472, and 505 genes contained premature termination codons in their internal sequences. Complete BUSCO scores ranged from 90.1% (AKIK2p1) to 91.4% (AKIK1p2).

Next, we compared the sequences of predicted genes with *MSE1*/*AoMYB35*/*AspTDF1*, which has been reported as the male-specific sex determinant gene in *A. officinalis.*^3–5^ Two genes, K1p1ch01g28074 and K1p2ch01g47751, identified in the genomes of the male line exhibited high sequence similarity with the query; however, none of the genes in the female genome assemblies showed significant sequence similarity with the query.

### 3.3. Genome sequence and structural variations between *Asparagus kiusianus* and *Asparagus officinalis*

Haplotype sequences within the male and female lines were compared. A total of 386,723 single nucleotide polymorphisms (SNPs) [transition (Ts)/transversion (Tv) ratio =3.0] and 46,007 insertions/deletions (indels) were identified between the two haplotypes of the male line (Table 4). On the other hand, 293,196 SNPs (Ts/Tv = 3.0) and 35,732 indels were identified between the two haplotype sequences of the female line (Table 4). The haplotype sequences of male and female lines were also compared, and 321,334 SNPs and 49,921 indels on average were identified across the four haplotype combinations (Table 4).

**Table 4:** Completeness of gene predictions, based on BUSCO assessment

	Within a line		Between male and female lines			
	AKIK1p1 vs AKIK1p2	AKIK2p1 vs AKIK2p2	AKIK1p1 vs AKIK2p1	AKIK1p1 vs AKIK2p2	AKIK1p2 vs AKIK2p1	AKIK1p2 vs AKIK2p2
Base substitutions	386,723	293,196	324,361	327,460	314,704	318,810
Ts/Tv ratio	3.0	3.0	2.9	2.9	2.9	2.9
1-bp indels	23,833	17,283	20,344	20,379	19,692	19,970
2-bp indels	5,799	4,477	5,722	5,763	5,645	5,691
3- to 49-bp indels	11,100	8,641	13,931	13,897	13,647	13,777
50- to 1000-bp indels	1,871	1,472	7,141	7,127	7,096	7,096
≥1000-bp indels	3,404	3,859	3,199	3,229	3,160	3,179

**Figure 2.**
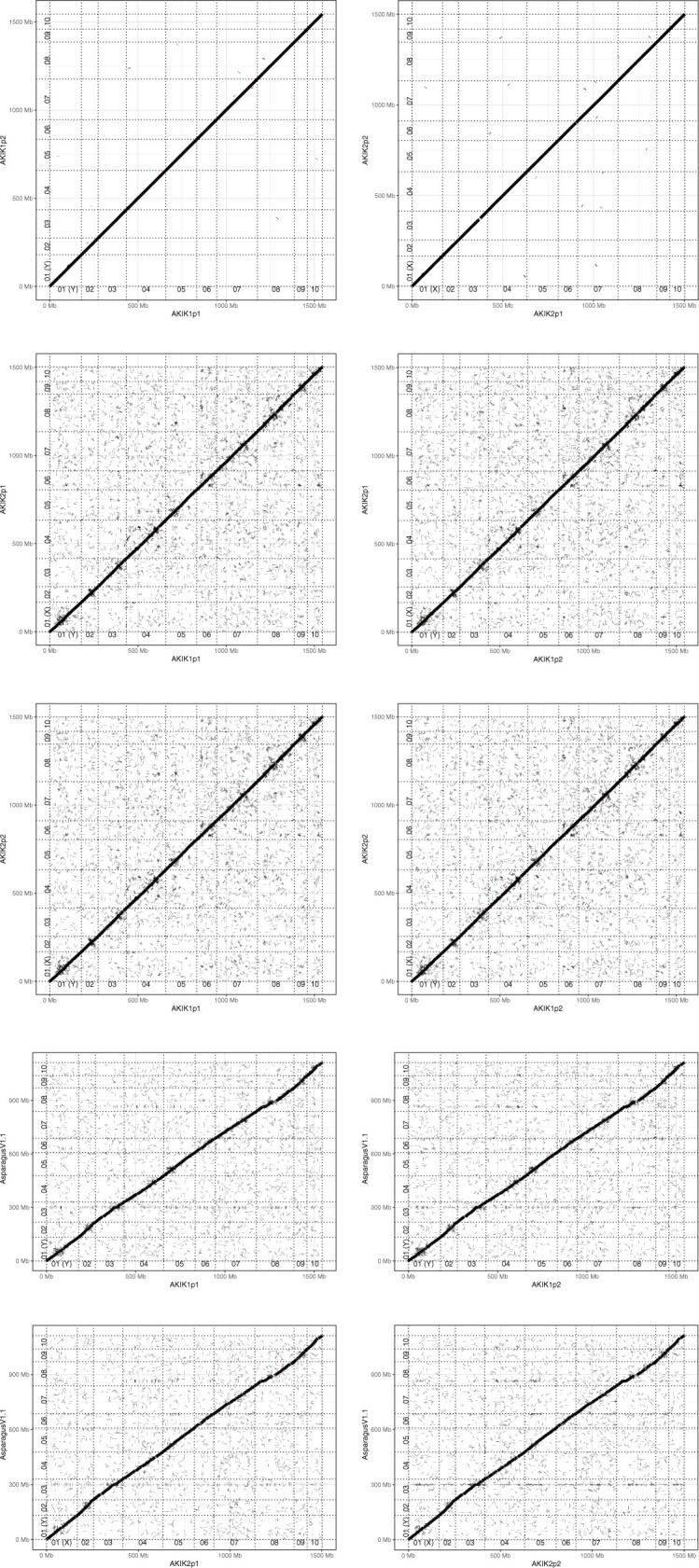
Comparative analysis of the genome sequence and structure of *A. kiusianus* and *A. officinalis*. Dots represent similarities between the genome sequence and structure of the two species. Genomes of the male and female lines of *A. kiusianus* are indicated as AKIK1 and AKIK2, respectively. Haplotype phases 1 and 2 are indicated as p1 and p2, respectively. AsparagusV1 indicates *A. officinalis* genome.

**Figure 3:**
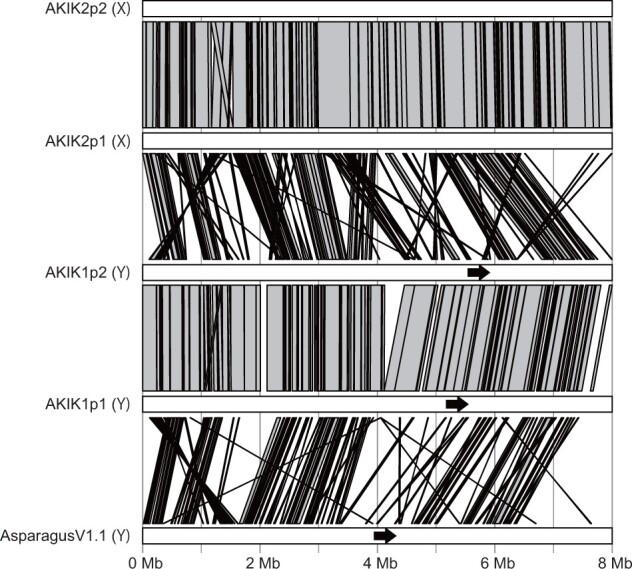
Comparison between the *M* locus in the Y-specific region on chromosome 1 (Y) of the male line and the corresponding region (X) of the female line. Grey bars indicate similarities in the genome sequence and structure. Black arrows indicate the position and orientation of *MSE1*/*AoMYB35*/*AspTDF1*.

While the chromosome structures were conserved within *A. kiusianus* lines and between *A. kiusianus* and *A. officinalis* (Fig. 2), genomic rearrangements were observed at the local level. For instance, at the sex-related region including the male-specific gene *MSE1*/*AoMYB35*/*AspTDF1* of *A. officinalis*, sequence collinearity was disrupted by inversions and translocations between the male and female lines (Fig. 3). Although sequence similarity was low between the male haplotypes of *A. kiusianus* and *A. officinalis*, sequence collinearity was moderately conserved (Fig. 3).

## 4. Conclusion and future perspectives

We present the chromosome-level haplotype-phased genome assemblies of the male and female lines of *A. kiusianus*, a wild relative of garden asparagus. The genome size of *A. kiusianus* was estimated to be ∼1.6 Gb (Fig. 1), which was 300 Mb larger than that of garden asparagus (ca. 1.3 Gb).^3^ This estimation was reflected in the difference between the assembly sizes of *A. kiusianus* (1.6 Gb) (Table 1) and garden asparagus (1.2 Gb). Of the 1.6 Gb assembly, 1.5 Gb could be aligned to the pseudomolecule sequence of asparagus, without any structural rearrangements (Fig. 2 and Table 2). Since we determined haplotype-phased genome sequences for the male and female lines of *A. kiusianus*, it was possible to compare the sequence and structure of the *M* locus between the Y-specific region of the male line and the corresponding region of the female line (Fig. 3). The result suggested dynamic genome rearrangements between the male and female lines, similar to that reported in jojoba,^9^ which might lead to presence/absence variation of the male-specific gene *MSE1*/*AoMYB35*/*AspTDF1* between the male and female lines.^3–5^

The genome of *A. kiusianus* harbours valuable genes that could be used for the breeding of elite garden asparagus cultivars. Because of cross-compatibility between the two species,^2^^,^^10^ important genetic loci, such as those imparting resistance to stem blight,^2^ which causes considerable production losses, could be transferred from *A. kiusianus* into garden asparagus. However, while DNA markers linked to the genes would facilitate the selection of disease-resistant lines in breeding programs, the genetic loci responsible for disease resistance have not been reported so far. The chromosome-level genome sequence of *A. kiusianus* presented in this study could serve as a reference for genetic mapping and the identification of resistance genes, as well as for transcriptome analysis and the determination of gene functions and mechanisms underlying the resistance and susceptible phenotypes.^6^^,^^7^

Although the plant genomics era started with the whole-genome sequencing of *Arabidopsis thaliana*,^11^ an undomesticated species, the advanced approaches of plant genomics have been applied more frequently to agronomically important crops rather than to wild plant species.^12^ Wild plants have the potential to accelerate the pace of breeding programs and to further the field of plant science.^13^^,^^14^ The genome sequence information of *A. kiusianus* generated in this study will help to reveal the genetic mechanisms underlying sexual differentiation in plants and will accelerate disease-resistance breeding in asparagus.

## Data availability

The genome sequence information generated in this study is available at Plant GARDEN (https://plantgarden.jp).

## Supplementary data

Supplementary data are available at DNARES online.

## Supplementary Material

dsac002_Supplementary_Data
